# Effect of water presence on choline chloride-2urea ionic liquid and coating platings from the hydrated ionic liquid

**DOI:** 10.1038/srep29225

**Published:** 2016-07-06

**Authors:** Cuiling Du, Binyuan Zhao, Xiao-Bo Chen, Nick Birbilis, Haiyan Yang

**Affiliations:** 1 State Key Laboratory of Metal Matrix Composites, Shanghai Jiaotong University, Shanghai 200240, PR China; 2 Department of Materials Science and Engineering, Monash University, Clayton, VIC 3800, Australia

## Abstract

In the present study, hygroscopicity of the choline chloride-urea (ChCl-2Urea) ionic liquid (IL) was confirmed through Karl-Fisher titration examination, indicating that the water content in the hydrated ChCl-2Urea IL was exposure-time dependent and could be tailored by simple heating treatment. The impact of the absorbed water on the properties of ChCl-2Urea IL, including viscosity, electrical conductivity, electrochemical window and chemical structure was investigated. The results show that water was able to dramatically reduce the viscosity and improve the conductivity, however, a broad electrochemical window could be persisted when the water content was below ~6 wt.%. These characteristics were beneficial for producing dense and compact coatings. Nickel (Ni) coatings plating from hydrated ChCl-2Urea IL, which was selected as an example to show the effect of water on the electroplating, displayed that a compact and corrosion-resistant Ni coating was plated from ChCl-2Urea IL containing 6 wt.% water doped with 400 mg/L NA at a moderate temperature. As verified by FTIR analysis, the intrinsic reason could be ascribed that water was likely linked with urea through strong hydrogen bond so that the water decomposition was suppressed during plating. Present study may provide a reference to prepare some similar water-stable ILs for plating.

Given their unique physical and chemical properties such as low vapor pressure, wide electrochemical windows, electrical conductivity, solubility for metal salts, ionic liquids (ILs) have proven to be a suitable alternatives for water in the electrodeposition of a number of metals, alloys and semiconductors^1^,^2^,^3^,^4^. In many cases, satisfactory quality of coatings or novel alloy compositions simply cannot be produced in aqueous solutions, requiring electroplating obtained from ILs due to the absence of water electrochemistry^5^,^6^,^7^,^8^.

However, water exists ubiquitously in ILs irrespective to their tendency to hydration. Even hydrophobic ILs, such as trimethyl-n-hexylammonium bis((trifluoromethyl)sulfonyl)amide (TMHA-Tf_2_N) and 1-butyl-3-methylimidazolium hexafluorophosphate (BMIM-PF_6_), were found to slightly host residual water even when special measures for dehydration have been taken^9^,^10^. In terms of ILs with high hydrophilicity, especially imidazolium and pyrrolidinium based ILs containing numerous halogen anions, they readily absorb moisture from the ambient environment. In general, the kinetic viscosity could be reduced and the electrical conductivity increased as a result of the presence of a certain amount of water^11^,^12^,^13^, but the additional water would notably decrease the electrochemical window^10^,^14^,^15^,^16^. Furthermore, absorbed water molecules could elicit decomposition of a number of ILs, leading to undesired side reactions and even corrosion of metals they contact^17^,^18^. Owing to the special precautions when dealing with nominally “dry” ILs, the extensive application of ILs in practical applications have been restricted to date^19^,^20^,^21^. As such, it is of great interest to investigate ILs with stability when exposed to air and/or moist environments.

A deep eutectic-based IL formed by mixing a quaternary ammonium salt, choline chloride (2-hydroxy-ethyltrimethyl-ammonium, ChCl), with a hydrogen-bond donor species urea with a molar ratio of 1:2 (ChCl-2Urea) has been known as water stable IL, and investigated in electroplating metal/alloy coatings, including Nickel (Ni), Zinc (Zn), Copper (Cu) and their alloys^22^,^23^,^24^,^25^, and even semiconductor and precursor films, such as CdS, CdSe, ZnS, Cu-In, Cu-In-Se and Cu-In-Ga-Se^26^,^27^. In our previous studies, it has been found that the as-prepared ChCl-2Urea IL initially containing 0.56 wt.% residual water would absorb more moisture from air when the cell was open to air during storage^28^,^29^. Interestingly, the additional water seems no negative effect on the Ni and its alloys coatings. To be the best of our knowledge, little work has focused on the effect of water presence on the properties of ChCl-2Urea IL and resulting metallic platings. In a review paper, it was stated that some unpublished work indicated that the presence of small amounts of water is actually beneficial to the morphology of the platings produced from eutectic-based IL^1^. One published work revealed that bright Cr deposites can be plated from a eutectic-based IL consisting of ChCl and CrCl_3_·6H_2_O containing 20 wt.% water, but the effect of water has not been quantitatively investigated^30^. It is important to unveil the intrinsic mechanisms about how the water affect the properties of ChCl-2Urea IL and the resultant coatings, and the results may provide a new guide to design and produce ILs with similar functionalities.

In the present study, hygroscopicity of the ChCl-2Urea IL was evaluated by water absorption as a function of the exposure time to the open air. Effect of water presence on the electrochemical behaviour, physical properties, including kinetic viscosity and electrical conductivity, and chemical structures of the hydrated ChCl-2Urea IL were systematically investigated by cyclic voltammetry (CV), rotary rheometer, conductivity meter and Fourier transform infrared spectroscopy (FTIR), respectively. To further elucidate the functionality of water on the formation of metallic electroplatings, electrodepositon of Ni upon Cu substrate was offered as an example to be investigated. The deposition was carried out from the ChCl-2Urea IL containing 0.2 M NiCl_2_ (ChCl-2Urea-(0.2 M)NiCl_2_ electrolyte) and various content of water, which was characterised by scanning electron microscopy (SEM) and the weight method for current efficiency measurement. The results indicate that, water greatly reduced the viscosity and improve the conductivity of the ChCl-2Urea IL but would not narrowed the electrochemical window when its content is below ~6 wt.%. This can be ascribed that the water was likely linked with urea through strong hydrogen bond, resulting in the suppression of hydrogen evolution. With the aid of the absorbed water, dense and corrosion-resistant Ni coatings can be produced at a moderate temperature of 318 K. Conceivably, the beneficial effect of water can be extended to other metal and alloys coatings plating from ChCl-2Urea IL.

## Results

### Hygroscopicity of the ChCl-2Urea ILs

**Figure 1:**
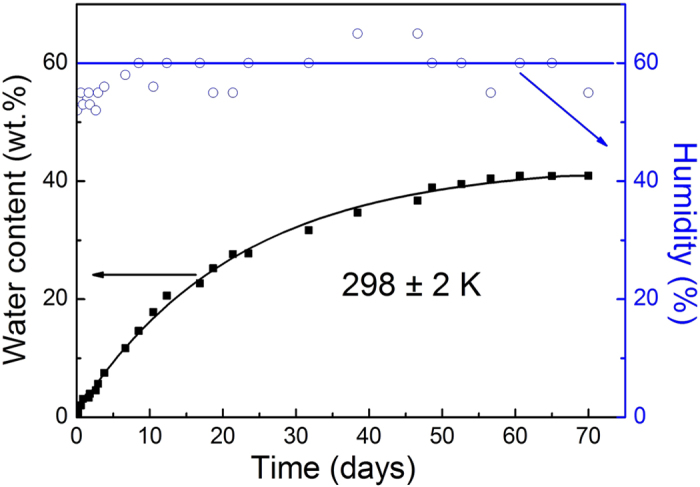
Water absorption as a function of the exposure time of the ChCl-2Urea IL to the open air. The points in open circles are the real-time humidity of the air. The temperature is maintained at 298 ± 2 K.

The as-prepared ChCl-2Urea IL contained 0.25 wt.% water even after a drying process conducted in vacuum oven at 353 K over 24 h (Table 1), indicating a high tendency of the ChCl-2Urea IL to water absorption, i.e. hygroscopic nature, when being exposed in open air. Figure 1 gives the plot of water absorption (wt.%) as a function of exposure time to air, revealing that the water absorption of ChCl-2Urea IL steadily increases with time and reaches a relative stable plateau of approximately 40 wt.% after in contact with air for 65 days. The temperature was maintained at 298 ± 2 K throughout the monitoring process and the real-time humidity of ambient environment was given in Fig. 1 to determine the environmental impact on the water absorption. The plot of water absorption represents a big slope during the exposure for the first hour and a water absorption rate of around 6.8 wt.% per day was identified, signaling that ChCl-2Urea IL had a dramatically high tendency to absorb water from air. And then the slope decreases rapidly down and a rate of around 3.4 wt.% per day was determined after 10 h, and then gradually reduces to the zero point after 65 days’ exposure to an environment containing a fluctuating humidity around 60%. It can be concluded that the water absorption to ChCl-2Urea IL is a time-dependent process regardless of the humidity in the surrounding environments. ChCl-2Urea IL absorbed moisture from open air quickly in the initial few hours’ exposure.

**Figure 2:**
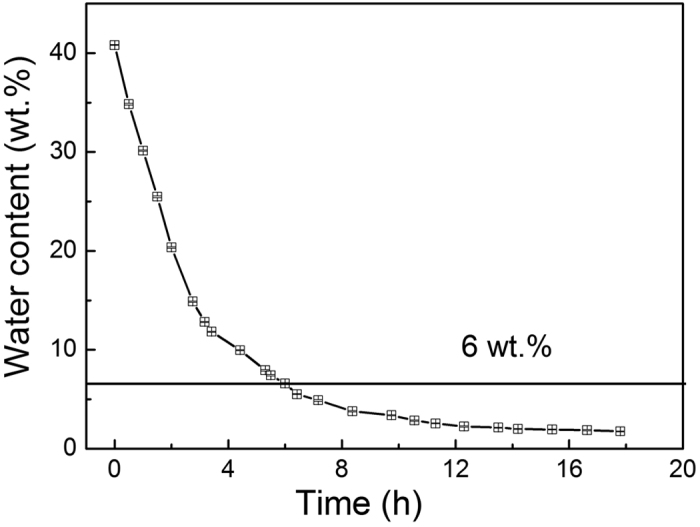
The variation of water content with heating time for the ChCh-2Urea-Water systems at 353 K. Error bars for the measured water content included and within the range of the data point.

**Figure 3:**
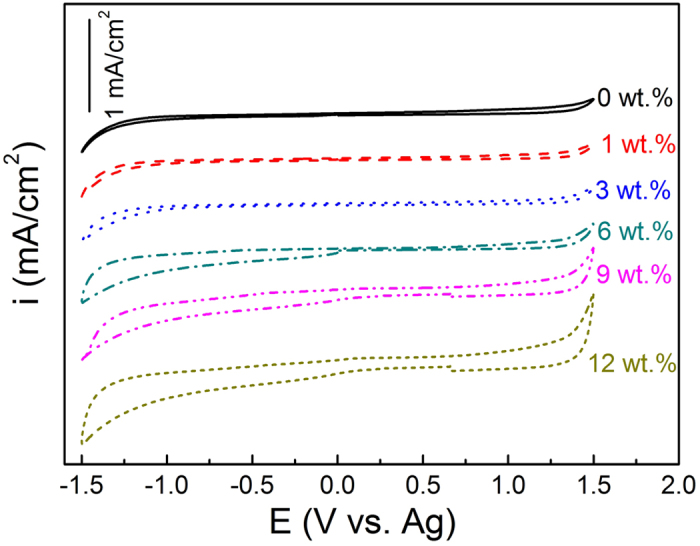
Cyclic voltammograms of the ChCl-2Urea-Water systems using a scan rate of 5 mV/s.

Given such importance of water content in the ChCl-2Urea IL regarding to their electrochemical functionalities as electrolyte for electroplating, it is highly desired to control the hydration degree of ChCl-2Urea ILs via simple techniques. Heat treatment was identified to be an effective way to eliminate water component from other IL-Water systems^31^. The validity of such heat treatment to regulate water absorption in the ChCh-2Urea-Water systems was confirmed through the measurement of water variation with time at 353 K (Fig. 2). It is evident that water content is rapidly decreased from ~40 wt.% to ~6 wt.% within 6 h, which is a favourable condition for electroplating purposes (verified by the CV tests presented in Fig. 3 and electroplatings in Section of Effect of water presence on the Ni electroplating). Continuing heat treatment beyond 6 h to 18 h could further get rid of water from the ChCl-2Urea ILs down to ~1.5 wt.%. Such a simple water controlling strategy could remove excessive water and transform the water-saturated ChCl-2Urea ILs into their original “dry” state for recycled uses, which could be a turning point for more extensive applications of ChCl-2Urea ILs with improved cost effectiveness.

### Effect of water presence on the electrochemical window of the ChCl-2Urea IL

Electrochemical window of an IL is essential in electrochemical application, in particular for electroplating. Previous studies unveiled that even a small quantity of water presence can dramatically decrease the electrochemical window, hence, many ILs are available electrolyte only when water absorption is strictly suppressed^14^,^20^,^32^.

The cyclic voltammogram measurements of the ChCl-2Urea-Water system reveal that the “dry” ChCl-2Urea IL exhibited a broad electrochemical window of 2.54 V, and which was limited by the cathodic potential at −1.14 V and anodic potential at 1.40 V (vs Ag). The cathodic and anodic limited potentials are determined by the reduction of Ch^+^ to triethylamine and the oxidation of Cl^−^ to Cl_2_ gas according to a latest report^33^. The electrochemical window of the ChCl-2Urea IL in the present study is comparable to that reported by Abbott (−1.2 to +1.25 V vs Ag on a Pt electrode)^34^. The slight difference is reasonable because the oxidation/reduction kinetics of the ILs considerably depends on the electrode materials and water content.

More importantly, the electrochemical window didn’t decrease substantially until the water content reached 6 wt.%, based on the fact that the anodic limited potential did not reduce and the movement of the cathodic limited potential in the positive direction was almost negligible (as illustrated in Fig. 3 and Table 2). When the water content increased to 6 wt.%, the anodic limited potential obviously moved towards the negative direction from 1.40 V to 1.22 V, and the cathodic limited potential dramatically moved along the positive direction from −1.10 V to −0.23 V, leading to a great decrease in the electrochemical window from 2.50 V to 1.45 V. The electrochemical window was further decreased with the increase in the water content, derived from the decrease in both anodic and cathodic limited potentials. The electrochemical window was decreased down to as narrow as ~1.00 V when the water content was more than 9 wt.% (Table 2).

Based on the above analysis, the water tolerance of the ChCl-2Urea IL can be determined as below 6 wt.% for electroplating applications, which is much higher than other existing ILs^14^,^15^. To our best knowledge, the reduction potential of Ni^2+^ is negative in various electroplating electrolytes^22^,^35^,^36^, therefore, only the ChCl-2Urea IL containing water below such a critical value (i.e. ≤6 wt.%) was considered in the following studies, given the fact that severe decomposition will occur during the course of plating once excessive water is present.

### Effect of water presence on the physical properties of the ChCl-2Urea IL

**Figure 4:**
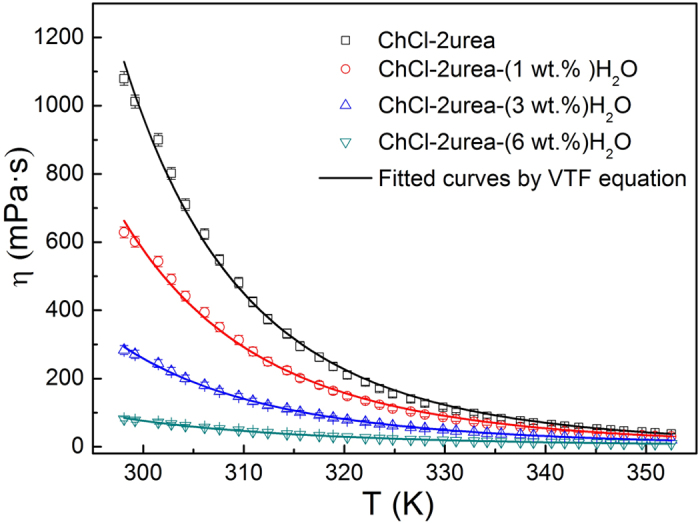
Viscosity of the ChCl-2Urea-Water systems as a function of temperature.

**Figure 5:**
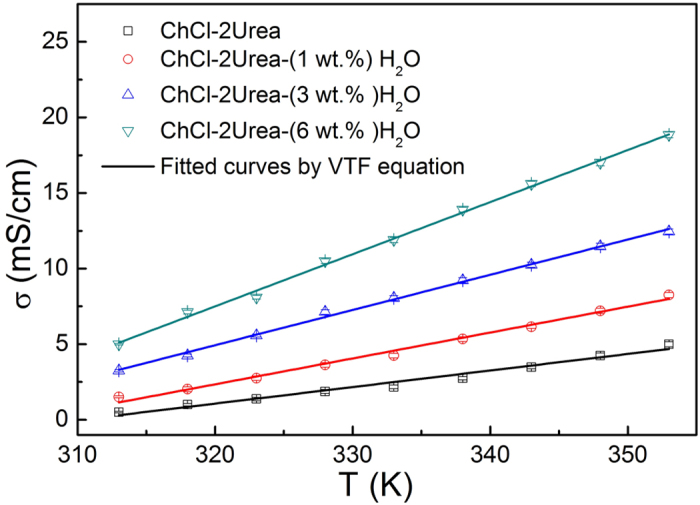
Conductivity of the ChCl-2Urea-Water system as a function of temperature.

Kinematic viscosity and electrical conductivity of the ChCl-2Urea-Water systems were carefully measured from 298 K to 353 K. The temperature dependence of kinematic viscosity and conductivity for the ChCl-2Urea-Water systems were depicted in Figs 4 and 5. They are very sensitive to the temperature, and the viscosity notably decreased while the conductivity increased with the increase of temperature. Such a temperature-dependent change is in accordance with previous results, but the kinematic viscosity of the “dry” ChCl-2Urea IL in the present study is relative higher and the electrical conductivity is lower than the reported values at elevated temperatures^18^. That may be attributed to the different content of water presenting in the ILs. According to the experiment details in the reference 18, the water has not been well controlled (less than 1 wt.%). Empirical equations such as Arrhenius equation, Vogel-Tammann-Fulcher (VTF) equation and polynomial have been widely used to describe the dependence of the viscosity and conductivity of ILs as a function of temperature^37^. It was found that the VTF equation applies well to the ChCl-2Urea-Water systems in the range of testing temperature, as shown in Figs 4 and 5.

It is evident both the viscosity and conductivity of the ChCl-2Urea-Water systems were sensitive to the content of water as well, as shown in Figs 4 and 5. As anticipated, with the increase of the water content, the viscosity was reduced while the conductivity was increased. For example, the viscosity was decreased by more than 13 times from 1080 mPa·s to 81 mPa·s at 298 K (Fig. 4), whereas the conductivity was improved by nearly one order of magnitude from 0.5 mS/cm to 4.9 mS/cm at 313 K (Fig. 5). However, the impact was impaired by elevated temperatures, implying that the electroplating could be performed at a lower temperature from a hydrated electrolyte than that required by a “dry” one.

The strong correlation between the viscosity (*η*) and conductivity (*σ*), as revealed in Figs 4 and 5, can be illustrated by the Stokes-Einstein equation and Nernst-Einstein equation, respectively.


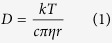



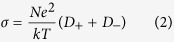


where *D* is the self-diffusion coefficient of the ionic species, *k* is the Boltzmann constant, *T* is the temperature, *c* is a constant determined by the boundary conditions, *r* is the Stokes radius of the ion, *N* is the Avogadro’s number, and e represents the electric charge. When ionic association takes place, the Nernst-Einstein equation should be modified by a dissociation factor, *α*.


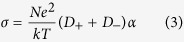


where α represents the degree of dissociation. Following equation can be deduced by substituting Eq. (1) into Eq. (3),


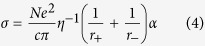


Therefore, ionic conductivity of the ChCl-2Urea-Water system is only dependent on the fluidity η^−1^, the Stokes radius of ion r and the degree of ionic dissociation α. It is assumed that the effect of water on r can be neglected in the linear region. So, the conductivity is mainly determined by the fluidity and degree of dissociation. Moreover, the degree of dissociation of the identical ILs does not change significantly with temperature^38^. Hence, a comparison of the conductivities at a fixed fluidity was expected to provide qualitative information about the degree of ionic dissociation of the ChCl-2Urea IL induced by hydration.

**Figure 6:**
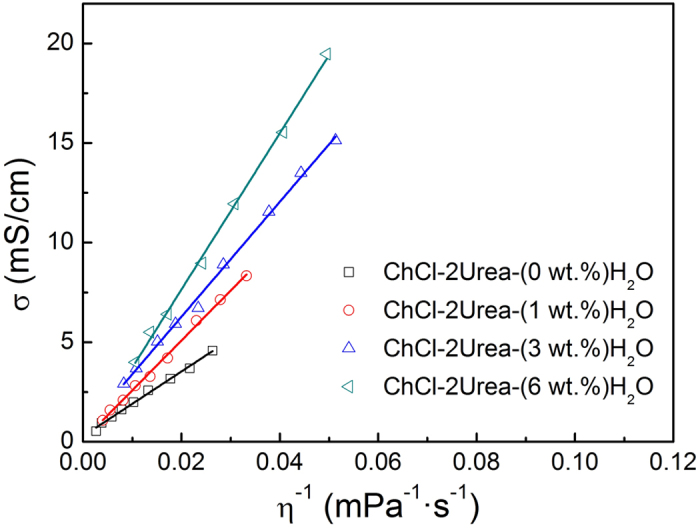
Conductivity of the ChCl-2Urea-Water system as a function of the inverse of viscosity.

Figure 6 illustrates the relationship of conductivity to the inverse of viscosity for the ChCl-2Urea-Water systems. As expected, the conductivity of all the systems was found to increase with the increase of fluidity, η^−1^. In addition, the conductivity was evidently increased with the increase in water content at a constant fluidity, indicating that the absorption of water into the “dry” ChCl-2Urea IL would lead to a significant promotion in ionic dissociation. A similar phenomenon has been found in other IL systems^13^,^39^.

So, the absorption of water can significantly decrease viscosity, and increase conductivity of the studied ChCl-2Urea IL, owing to the promotion of the ionic dissociation, which is beneficial for electroplating coatings with a high current efficiency at a relative lower temperature (refer to Section of Effect of water presence on the Ni electroplating).

### FTIR analysis

**Figure 7:**
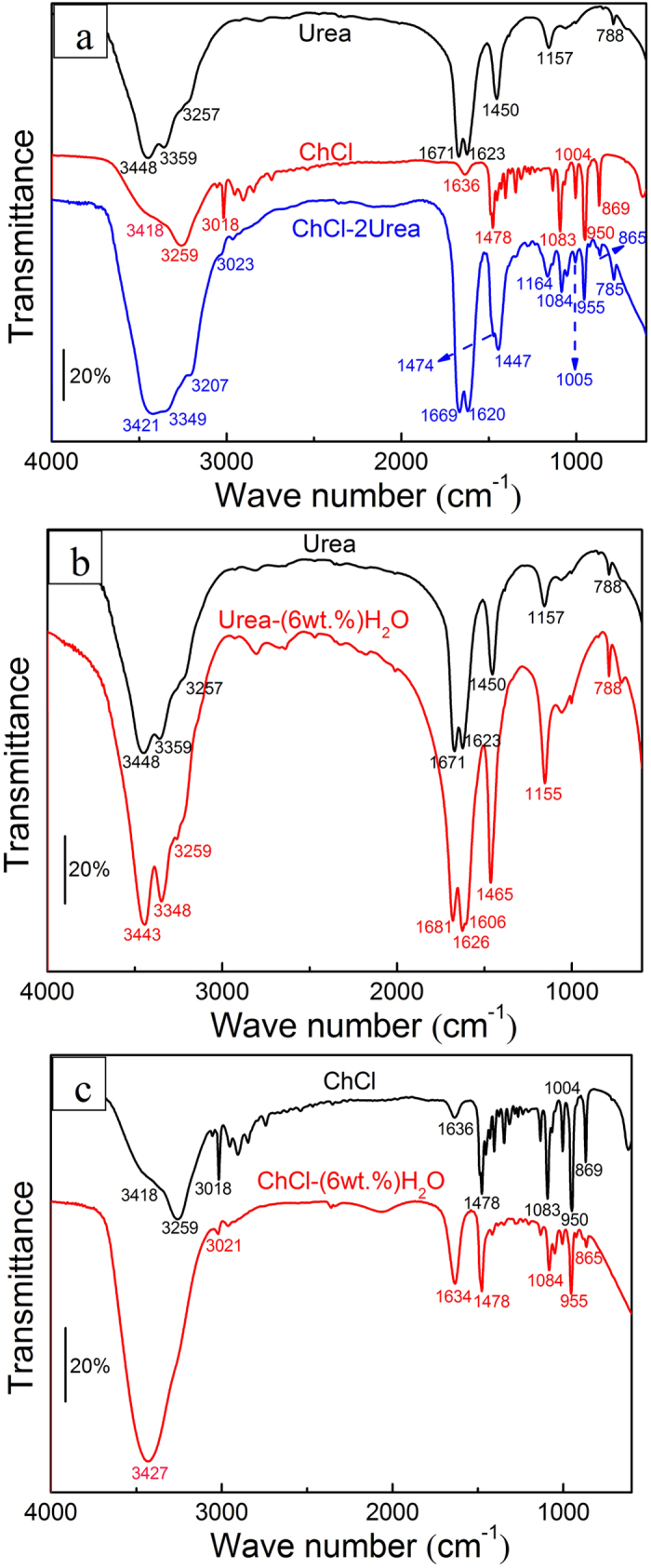
FTIR spectra of urea, ChCl and ChCl-2Urea IL with and without 6 wt.% H_2_O, (**a**) Dried Urea, ChCl and ChCl-2Urea IL, (**b**) Urea and Urea-(6 wt.%) H_2_O, and (**c**) ChCl and ChCl-(6 wt.%) H_2_O.

In order to determine the influence of water presence on the interactions between different terminal groups in the ChCl-2Urea IL, FTIR spectra of the ChCh-2Urea IL as well as the raw materials, including Urea and ChCl, before and after absorbing 6 wt.% water were recorded (Fig. 7), and the corresponding wave number (cm^−1^) of the vibrational modes and their associated terminal groups are listed in Table 3.

**Figure 8:**
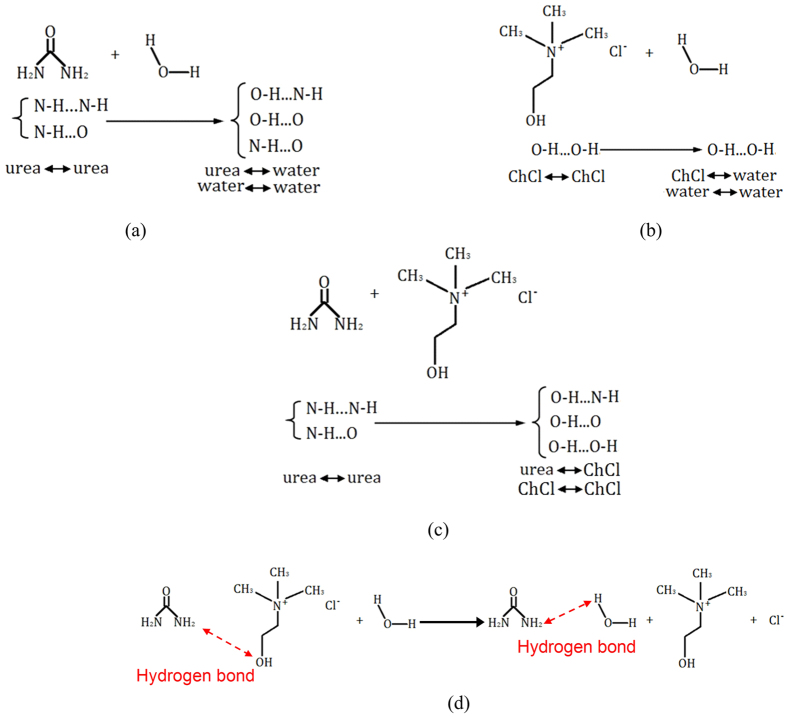
Postulated hydrogen bonds formed between urea, ChCl and water, (**a**) Urea-ChCl, (**b**) Urea-Water, (**c**) ChCl-Water and (**d**) effect of water on the hydrogen bond formed between Urea and ChCl.

Figure 7a shows the spectra of Urea, ChCl and ChCl-2Urea. It can be noted that, roughly, the characteristic spectrum of the ChCl-2Urea IL is an overlap of those of urea and ChCl. In addition, the bands associated to ChCl, such as ρ CH_3_, ρ CH_2_, ν_as_ CCO and δ CH appeared in spectrum of ChCl-2Urea. These results reveal that the structure of Ch^+^ was not destroyed in the ChCl-2Urea IL. Particularly, the absorption bands of Urea at 3448 cm^−1^ and 3359 cm^−1^, which can be ascribed to the stretching mode of -NH_2_ (ν_as_ NH_2_ and ν_s_ NH_2_), moved towards the lower wave number region to 3421 cm^−1^ and 3349 cm^−1^ and changed to broader bands. This could be ascribed to the forming of more hydrogen bonds between urea and ChCl^33^. The additional hydrogen bonds maybe exist as O-H···N-H, O-H···O and O-H···OH, as illustrated in Fig. 8a. However, as presented in Table 3, the water had a marginal effect on the vibration frequency of the groups in ChCl-2Urea IL by comparing the spectra before and after absorbing 6 wt.% water. Here, the spectrum of the ChCl-2Urea IL containing 6 wt.% water (ChCl-2Urea-(6 wt.%)H_2_O) was not shown for any duplication purpose. Nevertheless, some useful information could be deduced from the discussion on the effect of water on the raw materials.

Similar to the spectrum of ChCl-2Urea in Fig. 7a, the absorption bands of Urea ascribed to the stretching mode of -NH_2_ at 3448 cm^−1^ and 3359 cm^−1^ moved towards the lower wave number region due to absorbing water, as shown in Fig. 7b. Meanwhile, its absorption bands ascribed to the bending mode of -NH_2_ (δ_as_ NH_2_ and δ_s_ NH_2_) moved to the higher wave number area, and which can be attributed to the formation of more hydrogen bonds. The excess hydrogen bonds maybe exist as O-H···N-H, O-H···O and N-H···O, as depicted in Fig. 8b. In addition, a band at 1606 cm^−1^ ascribed to the bending vibration of -OH (δ_as_ OH) appeared given the co-existence of water as a result of hydration. Whereas, water have notable impact on the shape of the spectrum of ChCl. Two individual absorption bands of ChCl at 3418 cm^−1^ and 3259 cm^−1^, assigned to the stretching mode of -OH, were merged into a broad peak with a maximum peak value at 3427 cm^−1^ after absorbing water (shown in Fig. 7c), illustrating that the water existed as clusters or aggregates^31^. It can be expected that water molecules preferentially combined with other water molecules rather than ChCl according to their structural resemblance (Fig. 8c).

The structure of ChCl-2Urea IL has been investigated by FTIR but that particular didn’t focus on the water presence. Notably, FTIR spectrum of the Urea in the reference is in agreement with that of the Urea containing 6 wt.% water in the present study. It suggests that special cares should be taken regarding the analysis of ChCl-2Urea IL due to its high hygroscopicity^33^. Based on the above analysis, the water will preferentially combines with Urea molecules but not ChCl once ChCl-2Urea IL absorbs water from environment through hydrogen bond. Hence, the decomposition of water can be suppressed and the electrochemical window could be persisted due to the strong hydrogen bond, as revealed by the CV tests in Fig. 3. In the other hand, the fact that a fraction of the ChCl linked with Urea through hydrogen bond were replaced by water molecules will produce more free Ch^+^ and in turn result in a high ionic dissociation, as shown in Fig. 8d. This is due to the fact that water can interfere the coulombic force between Urea and ChCl through interacting with the -NH_2_ group, which may be the intrinsic reason why the ionic dissociation was promoted by water in Fig. 6. It has been proposed that hydrogen bond were formed preferentially with -NH_2_ when water was added into the similar protic ILs^40^,^41^,^42^.

### Effect of water presence on the Ni electroplating

**Figure 9:**
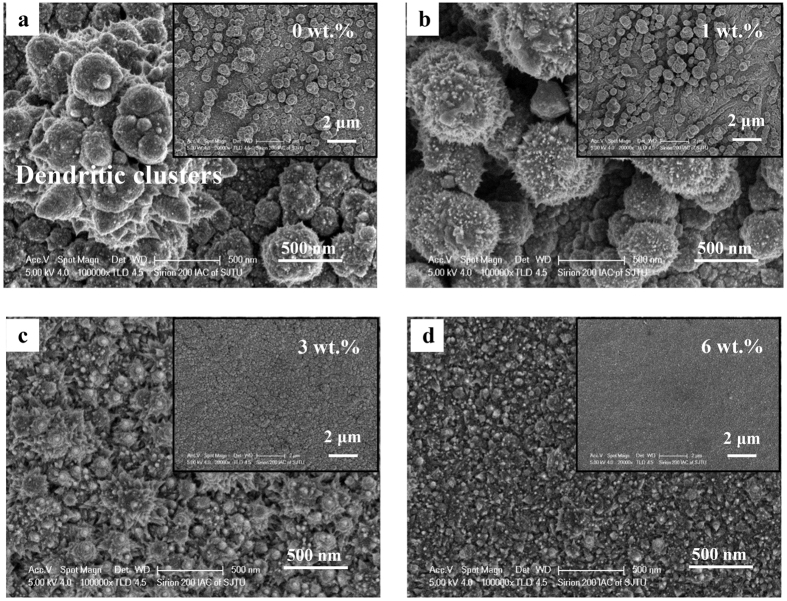
Micrographs of the Ni coatings electroplated from the ChCl-2Urea-(0.2 M)NiCl_2_ electrolytes containing (**a**) 0 wt.%, (**b**) 1 wt.%, (**c**) 3 wt.% and (**d**) 6 wt.% water at 318 K.

**Figure 10:**
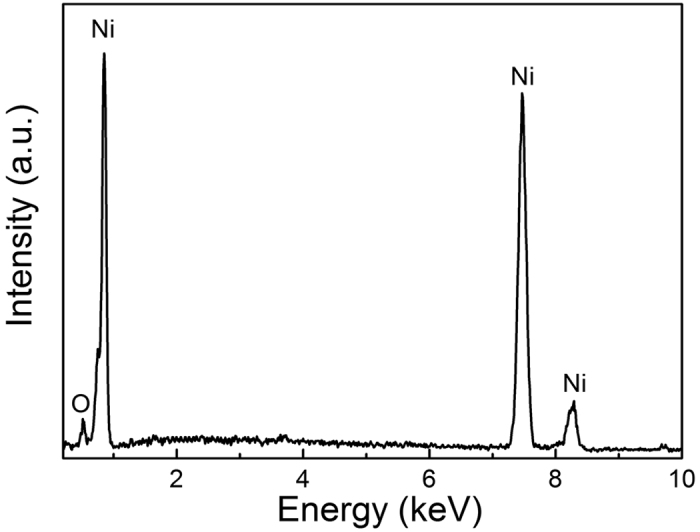
Typical EDXS pattern of the Ni coatings electroplated from the ChCl-2Urea-(0.2 M)NiCl_2_ electrolytes containing various content of water at 318 K.

Figure 9 displays the typical microscopic morphology of Ni coatings plated from ChCl-2Urea-(0.2 M)NiCl_2_ electrolytes at 318 K. It is evident that the surface morphology greatly depended on the water content. The deposits, from “dry” ChCl-2Urea-(0.2 M)NiCl_2_ electrolyte, present a rough and loose feature with irregular pyramid-like clusters with an uneven size distribution varying from 0.2 μm to 0.5 μm, and even dendritic growth was produced (Fig. 9a). The rough and loose surface gave rise to a dark grey appearance. A few dark particles that should be the Ni deposits detached from the coating were found in the bottom of the glass vessel, resulting in a low current efficiency of 47%. The powdery nature of such deposits and low current efficiency could be expected because they were plated under a high viscosity condition, and which generally led to irregular dendritic growth^43^. When the ChCl-2Urea-(0.2 M)NiCl_2_ electrolyte contained 1 wt.% water, the dendritic growth disappeared, leaving globular clusters (Fig. 9b). It should be attributed to the less viscous electrolyte. Furthermore, the current efficiency was markedly increased to 73%. The surface morphology and current efficiency were further improved when the water content was increased to 3 wt.%. The deposits became relatively dense and compact, and consisted of smaller cauliflower-like clusters. The micrograph taken at higher magnification reveals that these small clusters (*ca.* 100 nm) were composed of some much smaller and distinct nano-size dendritic crystals (Fig. 9c). However, the appearance was still in dark color although the current efficiency was further increased to 85%. As the water content was increased to 6 wt.%, the deposits exhibited a more uniform and compact surface with numerous finer pyramid-like clusters (*ca.* 30 nm, Fig. 9d), which produced a bright surface and high current efficiency of 98%. It is much higher than that in most aqueous Ni-electrodepositing system (where a cathodic efficiency is 65–75%) and close to that in a similar “dry” system but at an elevated temperature^29^. The positive effect of water on plated coatings can be attributed to the reduced viscosity of the ILs by water. Similar effect could be found in Cr platings produced from a very viscous IL system consisting of ChCl and CrCl_3_·6 H_2_O by comparing the provided SEM micrographs^30^,^44^,^45^. Importantly, in the present study, hydrogen evolution was suppressed during the electroplating process conducted in the electrolyte contains 6 wt.% water, which has been verified by a high cathodic current efficiency. Regardless of the differences in their morphologies, EDXS measurements indicated that the coatings were composed of mainly pure Ni except for low content of O due to surface oxidation, and were free of the ChCl-2Urea IL residual, as shown in Fig. 10.

**Figure 11:**
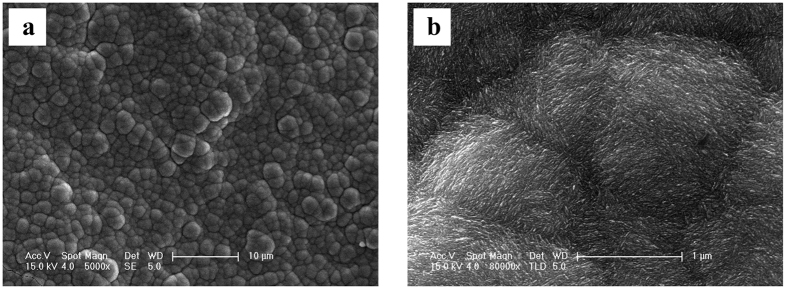
Micrographs of the Ni coatings electroplated from ChCl-2Urea-(0.2 M)NiCl_2_ electrolyte containing 6 wt.% water doped with 400 mg/L nicotinic acid at 318 K at (**a**) 5000× and (**b**) 80000× magnifications.

Though the above findings were promising, the Ni plating was not as compact as that plated from a similar ChCl-2Urea IL system doped with nicotinic acid (NA)^29^. As such, proper additive is still required for high quality electroplating from ChCl-2Urea-Water system. Figure 11 depicts the surface morphology of the Ni coating electroplated from the ChCl-2Urea-(0.2 M)NiCl_2_ electrolyte with 6 wt.% water doped with 400 mg/L NA. Spherical nodules, with sizes of 1~3 μm, were formed and these nodules consisted of nano-sized needle-like crystals, as revealed by the micrograph in higher magnification (Fig. 11b), leading to a compact and bright Ni coating.

**Figure 12:**
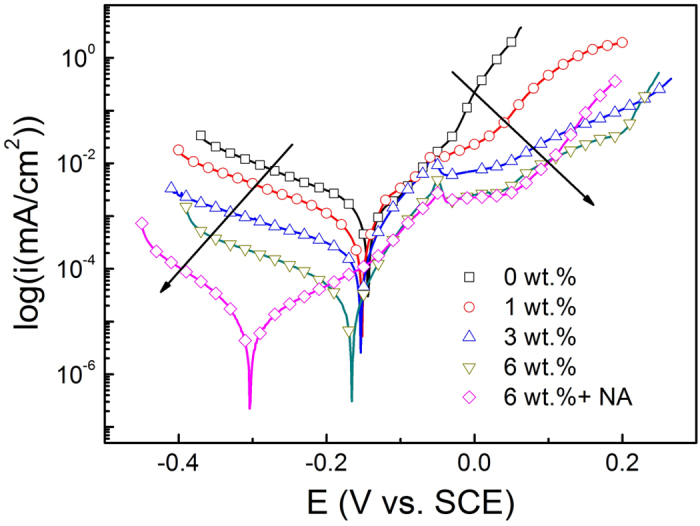
Potentiodynamic polarisation curves of the Ni coatings obtained from ChCl-2Urea-(0.2 M)NiCl_2_ electrolyte with various water content doped without and with 400 mg/L NA at 318 K. The black arrows indicate the change of anodic and cathodic kinetics as a result of water presence in the ChCl-2Urea IL electrolytes.

To evaluate the corrosion resistance of the Ni coatings, electrochemical behaviours were studied by potentiodynamic polarisation in 3.5 wt.% NaCl solution at 298 ± 2 K. Figure 12 illustrates the typical potentiodynamic polarisation curves for the Ni coatings electroplated from the ChCl-2Urea-(0.2 M)NiCl_2_ electrolyte with various hydration degrees and the estimated values of corrosion potential (E_corr_) and corrosion current density (*i*_corr_) through Tafel fitting were summarised in Table 4. As shown in Fig. 12, both the cathodic and anodic reaction kinetics of the coatings were suppressed with the increase in water content of the electrolytes, resulting in the decrease in corrosion current density (*i*_corr_) from 0.83 to 0.05 μA/cm^2^. The *i*_corr_ was further decreased to 0.01 μA/cm^2^ when the electrolyte was doped with 400 mg/L NA. The improved corrosion resistance could be attributed to the dense structure as illustrated in Figs 9 and 11. In addition, the E_corr_ moved towards more negative potential region, which could be ascribed to more passive nature of the yielded Ni coating whose compactness and surface coverage were gradually improved by water and NA absorbed/added into the ChCl-2Urea IL electrolytes.

## Conclusions

Hygroscopicity of ChCl-2Urea IL was studied via water absorption using the Karl-Fisher Titration method and it was shown that the quantity of water in the ChCl-2Urea IL closely depends on the duration time of exposure. Significant water absorption, of approximately 40 wt.% water, was obtained when ChCl-2Urea was exposed to ambient conditions for 65 days. Furthermore, the quantity of water in ChCl-2Urea was shown to be possible to tailor through simple heating. Cyclic voltammograms revealed that the original electrochemical window (spanning ~2.5 V) of ChCl-2Urea was retained when the water content was below ~6 wt.%. Water presence was able to greatly reduce viscosity and improve the conductivity of ChCl-2Urea. It was posited that the intrinsic reason was that the water were likely linked with urea through strong hydrogen bonds, generating the suppression of water decomposition and more free Ch^+^, *i.e.* the promotion of ionic dissociation, supported by FTIR analysis. A Ni plating which resulted in a more uniform, compact and corrosion-resistant could be prepared from hydrated ChCl-2Urea-(0.2 M)NiCl_2_ electrolyte containing 6 wt.% water doped with 400 mg/L NA at 318 K.

It is concluded that the “dry” ChCl-2Urea IL is not the most optimum alternative for aqueous media when conducting electroplating at moderate temperatures, such as 318 K. However, a small fraction of water absorbed into the ChCl-2Urea IL can remarkably improve the electrolyte system for electroplating by reducing viscosity and improving conductivity but will not narrow the electrochemical window. The work herein demonstrates possibilities in the preparation of compact coatings with a high current efficiency from hydrated ChCl-2Urea IL, though additive is still required for high quality electroplating.

## Methods

### Preparation of neat and 0.2 M NiCl_2_ containing ChCl-2Urea IL with various content of water

All chemicals in this work were of AR grade (99.9%) and provided by Sinopharm Chemical Reagent Co., Ltd. ChCl (HOC_2_H_4_N(CH_3_)_3_^+^Cl^−^) and urea were dried prior to use at 353 K in a vacuum oven (<133 Pa). Nickel chloride (NiCl_2_) and nicotinic acid (NA, C_6_H_5_NO_2_) were used as received. ChCl-2Urea IL was prepared by mixing pre-dried ChCl and urea with a molar ratio of 1:2 in a beaker. To minimise absorption of moisture from the atmosphere, pre-dried ChCl and urea chemicals were manually stirred by a glass rod for 30 s in open air, and then followed by a continuous stirring in a vacuum oven at 353 K for 30 min to achieve a crystal clear liquid. Hygroscopicity of the prepared ILs with exposure time to air was characterised through measurement of water content by Karl-Fisher Titration method (EM Science Aquastar V-200 Titrator). Afterwards, a simple heating treatment was applied to the ILs at 353 K to vary their water content. ChCl-2Urea IL containing absorbed water was named as ChCl-2Urea-Water system to reflect its hydrated nature hereafter.

ChCl-2Urea-Water system with different nominal content of water, including 0, 1, 3, 6, 9 and 12 wt.%, were selected for electrochemical window testing. The actual content of water in the ChCl-2Urea-Water systems measured by Karl-Fisher Titration method is listed in Table 1. Physical properties and chemical structures of all the ChCl-2Urea-Water systems were analysed except for the ones containing 9 and 12 wt.% water due to the fact that their reduced cathodic potential limits will incur severe self-decomposition during electroplating (see Fig. 3).

**Figure 13:**
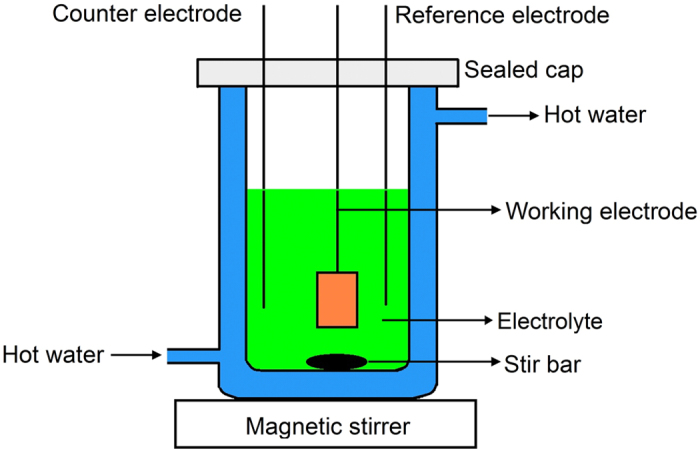
Schematic diagram of the setup for Ni electroplating.

To investigate the effect of water presence (say 0, 1, 3, 6 wt.%, respectively) on the electrochemical reduction of Ni^2+^ into metallic Ni coating, Ni electroplating process was conducted in the ChCl-2Urea-Water systems with an addition of 0.2 M NiCl_2_ (ChCl-2Urea-(0.2 M)NiCl_2_ electrolyte). After being sealed by Parafilm^®^ (PM-996, Pechiney, USA) in a glass vessel with a specifically designed double-wall structure, these mixtures were gently stirred until a transparent green liquid was obtained. The cavity between the double walls of the glass vessel was filled with circulated water to maintain the processing temperature at 318 K, which dominates the physical and electrochemical properties of ILs. The schematic diagram of the setup for electroplating of Ni is depicted in Fig. 13. The actual content of water in the ChCl-2Urea-(0.2 M)NiCl_2_ electrolyte is given in Table 1 as well.

It should be noted that the CV and electrical conductivity tests were carried out using the sealed setup as depicted in Fig. 13, to maintain the content of water and temperature. Karl-Fisher Titration results reveal that the content of water didn’t change a lot (<0.03 wt.%) during the experiments, indicating that the setup is valid.

### Electrochemical tests

CV examinations were performed to investigate the electrochemical window of the ChCl-2Urea-Water systems by an electrochemical workstation (CHI650D, Shanghai Chenhua Device Company, China) equipped with a typical three-electrode cell at a scan rate of 5 mV/s. A glassy carbon (GC) microelectrode (0.0707 cm^2^) and a platinum gauze (10 × 10 mm^2^) were used as working and counter electrode respectively, and a silver wire (99.9%, Alfa Aesar) inserted in a separated fritted glass tube containing the same IL as the bulk was used as reference electrode. All the electrodes were rinsed and dried before each measurement.

### Physical property measurements

Kinetic viscosity was measured using a rotary rheometer R/S-CPS + (Brookfield, USA) with a typical measurement system cone/plate (RC3-50^−1^, 0.008~2547 Pa·s). Viscosity curves were plotted using the Rheo3000 program at temperature varying from 298 K to 353 K. Electrical conductivity and its temperature dependence from 313 K to 353 K were determined by a conductivity meter (DDSJ-308 F, Shanghai Yidian Equipment Co., Ltd., China).

### FTIR characterisation

Chemical structures of the ChCl-2Urea ILs and the raw materials of ChCl and urea before and after absorbing water were analysed by a Nicolet 6700 FTIR (Thermo Fisher, MA, USA) where the ChCl-2Urea IL films were present in liquid state on dried KBr tablets. Scanning region was from 4000 cm^−1^ to 600 cm^−1^ with a resolution of 4 cm^−1^ and co-added for 64 scans. A background spectrum was also collected prior to each measurement and automatically subtracted from the acquired spectra.

### Electrodeposition and coating characterisation

Metallic Ni coatings were electrodeposited on Cu foil (99.98%, Aldrich) from ChCl-2Urea-(0.2 M)NiCl_2_ electrolyte by constant current mode at 318 K for 1 h, and the current density was consistently kept as 2.5 mA/cm^2^ for comparison. An identical set of counter (platinum gauze) and reference (Ag wire) electrodes as those used in the electrochemical tests were exploited. The working distance between the cathode and anode was maintained at 30 mm. Prior to electrodeposition, the Cu foil was cleaned in 65 wt.% HNO_3_ (40%, v/v) solution to remove oxides and contaminants from the surface. After electrodeposition, samples were thoroughly rinsed with distilled water and then dried in air.

The weight of electroplated Ni layer was measured through weighing the samples before and after being performed electroplating by an electronic balance (AL204, Mettler Toledo, China, ± 0.1 mg), and which was used to deduce the current efficiency based on the Faraday’s electrolysis law. In addition, the surface morphologies and elemental compositions of the Ni coatings were monitored using field emission scanning electron microscope (FE-SEM, SIRION200, FEI, America), equipped with energy dispersive X-ray spectrometry (EDXS).

Corrosion behaviours of the Ni eletroplatings were studied by potentiodynamic polarisation in 3.5 wt.% NaCl solution at 298 ± 2 K. A platimium gauze electrode and a saturated calomel electrode (SCE) were used as counter electrode and reference electrode, respectively. Prior to polarisation, the specimens were remained at the open circuit for 30 min to reach a stable state. Potentiodynamic polarisation plots were recorded from 250 mV below the OCP to and scanned upwards at a scanning rate of 1 mV/s.

## Additional Information

**How to cite this article**: Du, C. *et al*. Effect of water presence on choline chloride-2urea ionic liquid and coating platings from the hydrated ionic liquid. *Sci. Rep.* **6**, 29225; doi: 10.1038/srep29225 (2016).

## Figures and Tables

**Table 1:** Water content of ChCl-2Urea-Water systems as determined by Karl-Fisher titration examination and ChCl-2Urea-(0.2 M)NiCl_2_ electrolytes for Ni electroplating.

ILs	Water content (wt.%)	Electrolytes	Water content (wt.%)
ChCl-2Urea-0 wt.%	0.23 ± 0.01	ChCl-2Urea-(0.2 M)NiCl_2_-0 wt.%	0.25 ± 0.02
ChCl-2Urea-1 wt.%	1.10 ± 0.03	ChCl-2Urea-(0.2 M)NiCl_2_-1 wt.%	1.26 ± 0.03
ChCl-2Urea-3 wt.%	3.05 ± 0.05	ChCl-2Urea-(0.2 M)NiCl_2_-3 wt.%	2.99 ± 0.04
ChCl-2Urea-6 wt.%	5.99 ± 0.03	ChCl-2Urea-(0.2 M)NiCl_2_-6 wt.%	5.99 ± 0.02
ChCl-2Urea-9 wt.%	9.11 ± 0.04		
ChCl-2Urea-12 wt.%	11.9 ± 0.04		

**Table 2:** Electrochemical parameters, including cathodic limited potentials, anodic limited potentials and electrochemical windows, of the various ChCl-2Urea-Water systems obtained from cyclic voltammogram measurements.

Nominal H_2_O content (wt.%)	0	1	3	6	9	12
Cathodic limited potential (V vs Ag)	−1.14	−1.13	−1.11	−0.23	0.11	0.18
Anodic limited potential (V vs Ag)	1.40	1.40	1.40	1.22	0.95	0.95
Electrochemical window (V vs Ag)	2.54	2.53	2.50	1.45	1.06	1.03

**Table 3:** Wave numbers and their assignments obtained from FTIR spectra.

Urea	Urea-6 wt.%H_2_O	ChCl	ChCl-6 wt.%H_2_O	ChCl-2Urea	ChCl-2Urea-6 wt.%H_2_O	Assignments
3448	3443			3421	3421	ν_as_ NH_2_
		3418	3427			ν_as_ OH
3359	3348			3349	3349	ν_s_ NH_2_
3257	3259					ν_as_ NH_2_
		3259				ν_as_ OH
				3207	3207	ν_as_ OH
		3018	3021	3023	3022	δ_as_ OH
1671	1681			1669	1667	δ_s_ NH_2_
	1645	1636	1634			δ_as_ OH
1623	1626			1620	1621	δ_as_ NH_2_
	1606					δ_as_ OH
		1478	1478	1474	1472	ρ CH_3_
1450	1465			1446	1449	ρ_s_ NH_2_
1157	1155			1164	1163	ν_as_ CN
		1083	1084	1084	1084	ρ CH_2_
		1004	1006	1005	1006	ν C-O
		950	955	955	955	ν_as_ CCO
		869	865	865	865	ν_s_ N-CH_3_
788	788			785	785	ω C = O

**Table 4:** Parameters obtained for Ni coatings electroplated from ChCl-2Urea-(0.2 M)NiCl_2_ electrolytes with various water contents doped with or without 400 mg/L NA at 318 K and pure Ni sheet, estimated through Tafel fitting of the polarisation curves obtained in 3.5 wt.% NaCl solution.

Nominal water content in the electrolytes (wt.%)	0	1	3	6	6 (with 400 mg/L NA)
E_corr_ (mV vs.VSCE)	−1440 ± 12	−1520 ± 9	−1540 ± 14	−1660 ± 10	−304 ± 7
i_corr_ (μA/cm^2^)	0.83 ± 0.05	0.63 ± 0.07	0.23 ± 0.04	0.05 ± 0.01	0.01 ± 0
